# Case Report: Neonatal COL1A1-related osteogenesis imperfecta recognized after multiple fractures in an infant admitted for jaundice

**DOI:** 10.3389/fped.2026.1962700

**Published:** 2026-10-30

**Authors:** Shuxia Gui, Jingxian Gong, Xiaoqin Pi, Jing Zhou, Lingkong Zeng, Wenhao Yuan

**Affiliations:** 1 Department of Neonatology, Wuhan Children's Hospital (Wuhan Maternal and Child Health Hospital), Tongji Medical College, Huazhong University of Science & Technology, Wuhan, Hubei, China; 2 Department of Radiology, Wuhan Children's Hospital (Wuhan Maternal and Child Health Hospital), Tongji Medical College, Huazhong University of Science & Technology, Wuhan, Hubei, China

**Keywords:** COL1A1 variant, diagnostic challenge, multiple fractures, neonate, osteogenesis imperfecta, whole-exome sequencing

## Abstract

**Background:**

Osteogenesis imperfecta (OI) is a rare inherited skeletal fragility disorder characterized by impaired bone strength and increased susceptibility to fractures. Most genetically confirmed cases are associated with pathogenic variants affecting type I collagen, particularly COL1A1 and COL1A2. Severe neonatal OI may present with multiple fractures, skeletal deformities, and respiratory complications; however, early diagnosis remains challenging because classical clinical manifestations may be absent during the neonatal period.

**Case presentation:**

We report a term male neonate with COL1A1-related osteogenesis imperfecta associated with an apparently *de novo* pathogenic variant in COL1A1. The infant was born at 38 ^+^ ^4^ weeks of gestation by elective cesarean delivery with a birth weight of 3040 g. He was initially admitted to the neonatal intensive care unit because of progressive jaundice on day 3 of life. No obvious skeletal abnormalities, limb deformities, or apparent blue sclerae were identified during the initial examination. On day 5 after birth, swelling and reduced spontaneous movement of the right lower extremity were observed. Radiological evaluation identified right femoral, radial, clavicular, and multiple rib fractures. The timing of fracture occurrence remained uncertain. Laboratory investigations showed normal serum calcium, phosphorus, alkaline phosphatase, parathyroid hormone, and vitamin D levels, making a major mineral metabolism disturbance less likely. Subsequent trio whole-exome sequencing identified a heterozygous apparently *de novo* missense variant in COL1A1 (NM_000088.4:c.581G > A, p.Gly194Asp), located within the triple-helical domain of type I collagen. The laboratory classified the variant as pathogenic according to the American College of Medical Genetics and Genomics/Association for Molecular Pathology criteria. The patient received multidisciplinary management, including fracture stabilization, protective nursing strategies, orthopedic intervention, and genetic counseling. Early follow-up demonstrated progressive fracture healing with callus formation; clinical severity classification requires longer follow-up.

**Conclusion:**

This case highlights that neonatal OI may initially present with nonspecific clinical manifestations and without classical phenotypic features. Unexplained fractures in neonates should prompt consideration of inherited skeletal fragility disorders. Early molecular diagnosis is essential for confirmation of diagnosis, genetic counseling, prevention of secondary injury, and individualized multidisciplinary management.

## 1 Introduction

Osteogenesis imperfecta (OI) is a rare and clinically heterogeneous group of inherited connective tissue disorders characterized primarily by increased bone fragility, recurrent fractures, and variable extraskeletal manifestations. Most genetically confirmed OI cases are associated with pathogenic variants affecting type I collagen, particularly COL1A1 and COL1A2, which encode the *α*1 and *α*2 chains of type I collagen, the major structural component of the bone extracellular matrix (ECM). The clinical spectrum of OI is highly variable, ranging from mild forms with limited fracture susceptibility to severe neonatal disease characterized by multiple fractures, progressive skeletal deformities, and respiratory compromise (1, 2). The classical Sillence classification system categorized OI into four major clinical types according to severity, radiographic characteristics, and inheritance patterns. Among these, type III OI represents a severe, progressively deforming phenotype associated with recurrent fractures, short stature, and progressive skeletal abnormalities (3, 4). However, subsequent molecular studies have demonstrated substantial genotype–phenotype variability, and clinical severity cannot always be accurately predicted solely from the identified genetic variant (4–6).

Diagnosis of neonatal OI remains particularly challenging because characteristic manifestations may not be apparent immediately after birth. Although severe cases may present prenatally with fractures, limb deformities, or abnormal skeletal mineralization, some affected neonates may initially appear clinically normal. Classical findings such as blue sclerae, short stature, or a positive family history may be absent, while a *de novo* variant can explain a negative family history (4–6). The molecular pathogenesis of OI is primarily related to abnormalities in type I collagen synthesis, assembly, or stability. The collagen triple-helical domain contains a highly conserved Gly-X-Y repeat sequence, and glycine residues are essential for proper triple-helix formation because of their unique small molecular structure. Substitution of glycine residues within this region disrupts collagen folding and stability and is strongly associated with moderate-to-severe OI phenotypes (1, 7, 8).

With the rapid development of next-generation sequencing technologies, molecular genetic testing has become an essential component of the diagnostic evaluation of suspected OI. Genetic confirmation establishes the diagnosis, facilitates genotype–phenotype correlation, enables recurrence risk assessment, and provides important information for individualized clinical management (5, 6). Interpretation of sequence variants should follow standardized criteria established by the American College of Medical Genetics and Genomics and the Association for Molecular Pathology (ACMG/AMP) (9). Although numerous OI-associated variants have been reported, neonatal patients presenting mainly with nonspecific symptoms and lacking classical skeletal manifestations remain diagnostically challenging. Here, we report a term neonate with COL1A1-related OI initially admitted for neonatal jaundice, in whom multiple fractures subsequently led to the diagnosis of an apparently *de novo* COL1A1 c.581G > A (p.Gly194Asp) variant. This case emphasizes the importance of considering inherited skeletal fragility disorders in neonates with unexplained fractures, even in the absence of typical clinical features (3–6).

## 2 Case presentation

### 2.1 Patient information and perinatal history

A male neonate was born at 38 ^+^ ^4^ weeks of gestation by elective cesarean delivery. His birth weight was 3040 g; Apgar scores were not recorded. The pregnancy was reportedly uncomplicated, and routine prenatal ultrasonography did not reveal obvious skeletal abnormalities, limb deformities, abnormal mineralization, or intrauterine fractures. The parents were non-consanguineous and clinically healthy. Neither parent had a history of recurrent fractures, short stature, skeletal deformities, dentin abnormalities, or known genetic disorders. There was no family history suggestive of inherited skeletal fragility disorders. After birth, the infant was considered clinically stable. Routine neonatal examination did not identify obvious abnormalities of the limbs, thorax, or skull. In particular, no apparent features suggestive of osteogenesis imperfecta (OI), including marked skeletal deformity, limb shortening, or prominent blue sclerae, were observed. A normal initial examination and unremarkable prenatal ultrasound do not establish that fractures were absent at birth. The family history included an older sister and two previous pregnancy losses, without other affected relatives.

### 2.2 Initial presentation and admission to the NICU

On the third day after birth (May 30, 2026), the infant was transferred to the neonatal intensive care unit (NICU) because of progressive jaundice. At admission, physical examination revealed neonatal jaundice without hepatosplenomegaly or other systemic abnormalities. The infant had stable vital signs, acceptable respiratory status, and no obvious limitation of limb movement.

Laboratory evaluation demonstrated hyperbilirubinemia compatible with neonatal jaundice. Phototherapy was initiated, and bilirubin levels gradually improved. Because no skeletal abnormalities were identified during the initial assessment, inherited skeletal disorders were not initially suspected. No obvious characteristic facial features or blue sclerae were noted at that time (Figures 1A,B). Jaundice was the reason for admission; a causal association with OI or fracture-related hemolysis was not established.

**Figure 1:**
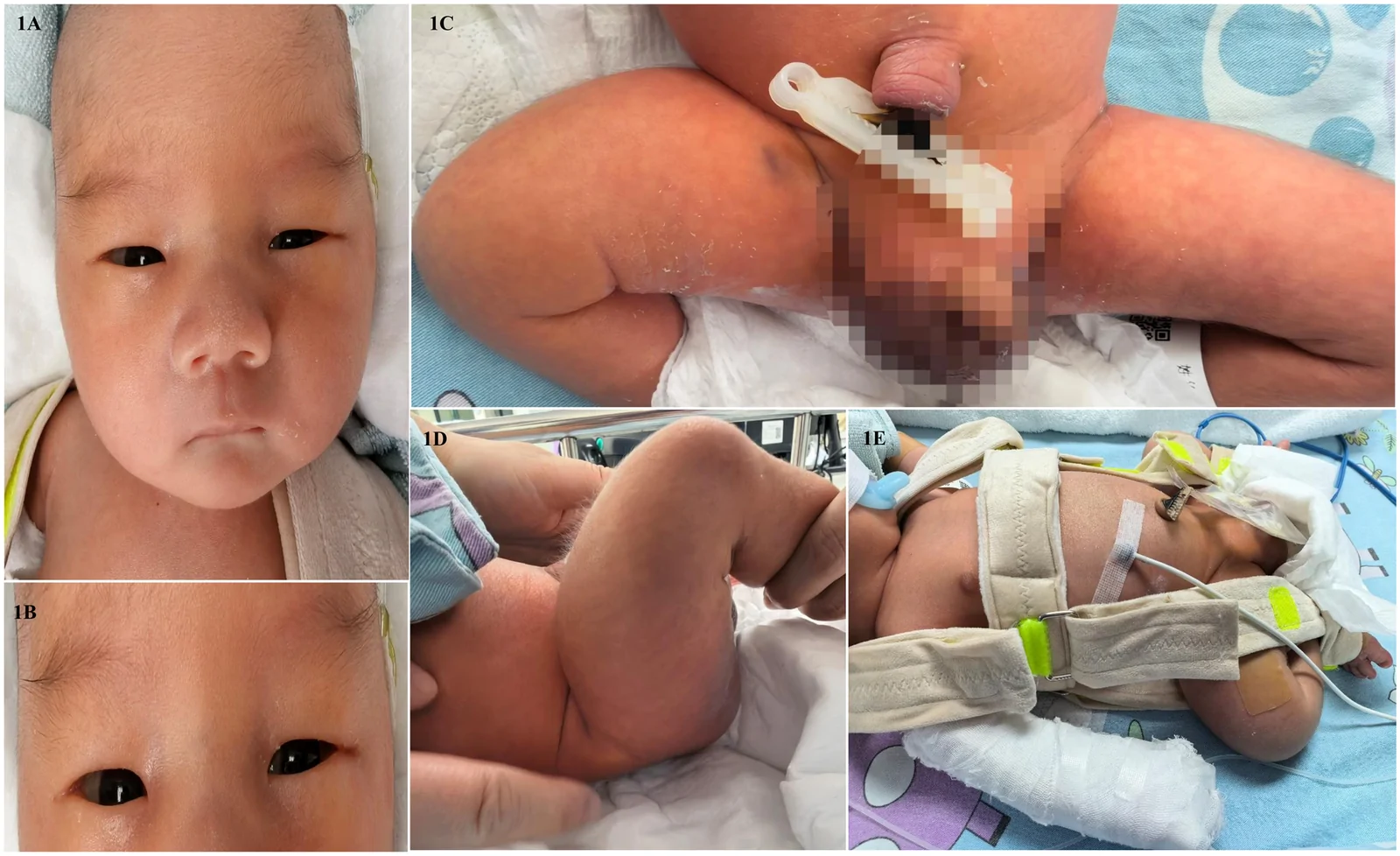
Clinical presentation and initial diagnostic findings of the neonate with COL1A1-related osteogenesis imperfecta. **(A)** Facial appearance of the patient during the early neonatal period. No obvious craniofacial abnormalities or characteristic features of osteogenesis imperfecta were observed. **(B)** Close-up view of the ocular region showing no prominent blue sclerae at initial examination. **(C)** Clinical manifestation after symptom onset, showing swelling and decreased spontaneous movement of the right lower extremity associated with the femoral fracture. **(D)** Bedside examination showing apparent angulation of the right upper limb compared with the contralateral side. **(E)** Orthopedic management after diagnosis, showing stabilization of the femoral fracture using a Pavlik harness.

### 2.3 Development of fracture-related symptoms during hospitalization

On June 1, 2026 (five days after birth), right lower limb swelling and reduced spontaneous movement (Figure 1C) prompted bedside ultrasonography. The ultrasound report at 16:48 described femoral cortical discontinuity, soft-tissue swelling, and a possible intermuscular hematoma (Supplementary Figure S1). Four-limb radiographs acquired at 18:20 identified right femoral shaft, right radial, right clavicular, and left second to sixth rib fractures, with displacement. A chest radiograph acquired at 18:23 also demonstrated the clavicular and rib fractures. Left proximal humeral cortical irregularity was described as a possible incomplete fracture and remained unconfirmed. The June 2 right upper limb radiograph was a follow-up study, not the first detection of the radial fracture. Skull and targeted follow-up imaging were obtained, but dedicated hand and foot views were not performed; a standardized complete skeletal survey was not documented. No baseline imaging established intact bones, so fracture occurrence cannot be dated from symptom recognition. Bedside examination also showed apparent angulation of the right upper limb (Figure 1D).

### 2.4 Differential diagnosis and laboratory evaluation

The differential diagnosis of neonatal multiple fractures included: 1) Birth-related traumatic fractures; 2) Metabolic bone disorders, including disorders of calcium-phosphate metabolism and neonatal bone mineralization abnormalities; 3) Non-accidental injury; 4) Inherited skeletal fragility disorders, particularly osteogenesis imperfecta.

### 2.5 Birth trauma

Birth trauma was considered because neonatal fractures may occur during delivery (10, 11). However, this infant was delivered by elective cesarean section, and no history of difficult labor or mechanical injury was identified. Furthermore, the distribution and number of fractures, particularly the presence of multiple rib fractures and fractures involving different skeletal sites, were less consistent with isolated birth-related trauma. Elective cesarean delivery does not exclude birth-related injury, and the timing of fracture occurrence remains uncertain.

### 2.6 Non-accidental injury

Although non-accidental injury is uncommon in the immediate neonatal period, it remains an important consideration in infants with unexplained fractures. A careful evaluation of fracture patterns, clinical history, and social circumstances is required (10, 11). The molecular finding supported inherited skeletal fragility in this patient, but genetic OI does not independently exclude coexisting traumatic or inflicted injury.

### 2.7 Metabolic bone disorders

Laboratory evaluation was performed to assess possible metabolic causes of increased bone fragility. Serum calcium was 2.2 mmol/L (reference range: 2.1–2.8 mmol/L), serum phosphorus 1.86 mmol/L (1.6–2.51 mmol/L), alkaline phosphatase (ALP) 281 U/L (48–406 U/L), parathyroid hormone (PTH) 31.2 pg/mL (14–72 pg/mL), and 25-hydroxyvitamin D [25(OH)D] 34.41 ng/mL (>30 ng/mL). All results were within the corresponding laboratory reference ranges (Supplementary Table S1). These findings did not support a major mineral metabolism disturbance as the primary cause of fractures (12).

### 2.8 Radiological findings

Radiological examination played a critical role in establishing the diagnosis. The June 1 radiographs demonstrated a displaced and angulated right femoral shaft fracture (Figure 2A), a displaced right radial fracture, a right clavicular fracture, and left second to sixth rib fractures (Figure 2B). Suspected left proximal humeral involvement remained unconfirmed. The presence of fractures affecting multiple skeletal regions suggested an underlying systemic disorder of bone fragility. Although severe neonatal OI may be associated with generalized osteopenia or obvious skeletal deformities, these findings were not prominent during the early neonatal period in this patient (1, 4, 13). Follow-up radiographs demonstrated femoral callus formation and residual angulation (Figure 2C). The combination of multiple fractures, normal biochemical markers, and clinical suspicion of inherited skeletal fragility prompted molecular genetic testing.

**Figure 2:**
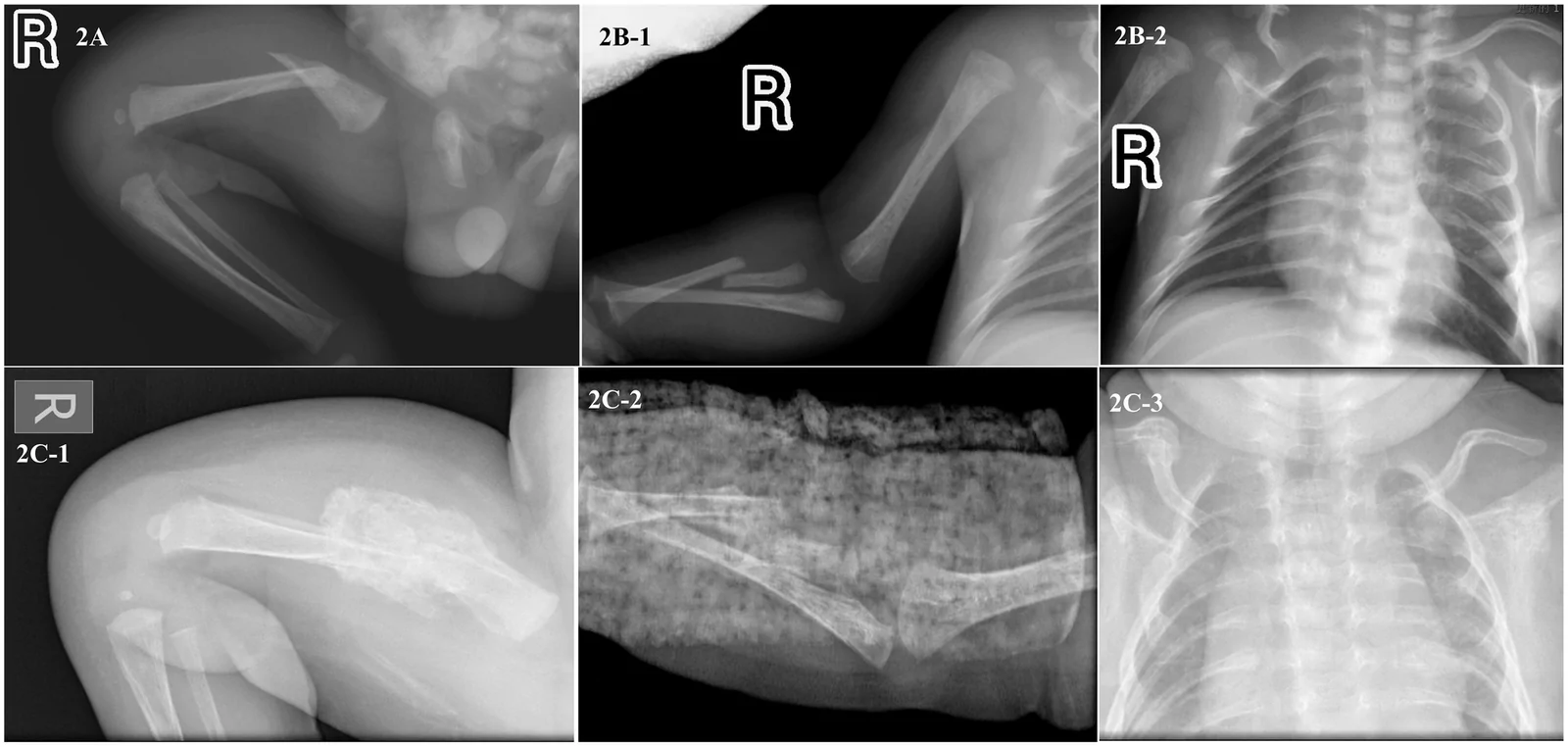
Radiological evolution and fracture healing during follow-up. **(A)** Right femoral shaft fracture with displacement and angulation. **(B)** Additional imaging showing right radial, right clavicular, and left second to sixth rib fractures; these fractures were identified on June 1, 2026. **(C)** Follow-up radiographs approximately one month after discharge showing callus formation, progressive healing of the femoral fracture, and mild residual angulation.

### 2.9 Genetic diagnosis

Genetic testing was recommended during the June 2 multidisciplinary consultation; results remained pending at discharge. Trio whole-exome sequencing (WES) was performed using genomic DNA obtained from the patient and both parents. Genetic analysis subsequently identified a heterozygous missense variant in COL1A1: NM_000088.4:c.581G > A (p.Gly194Asp) (Figure 3; Supplementary Table S1). The variant affects a highly conserved glycine residue located within the collagen triple-helical domain of type I collagen (1, 7, 8). Sanger sequencing detected the variant in the patient but not either parent, supporting an apparently *de novo* occurrence. The laboratory classified the variant as pathogenic according to the American College of Medical Genetics and Genomics/Association for Molecular Pathology (ACMG/AMP) criteria (9), with reported evidence codes PS2, PM1, PM2, PP2, and PP3. Together with the clinical phenotype of multiple neonatal fractures, these findings supported COL1A1-related OI. The Sillence subtype remains unassigned because progressive skeletal deformity has not been demonstrated (4).

**Figure 3:**
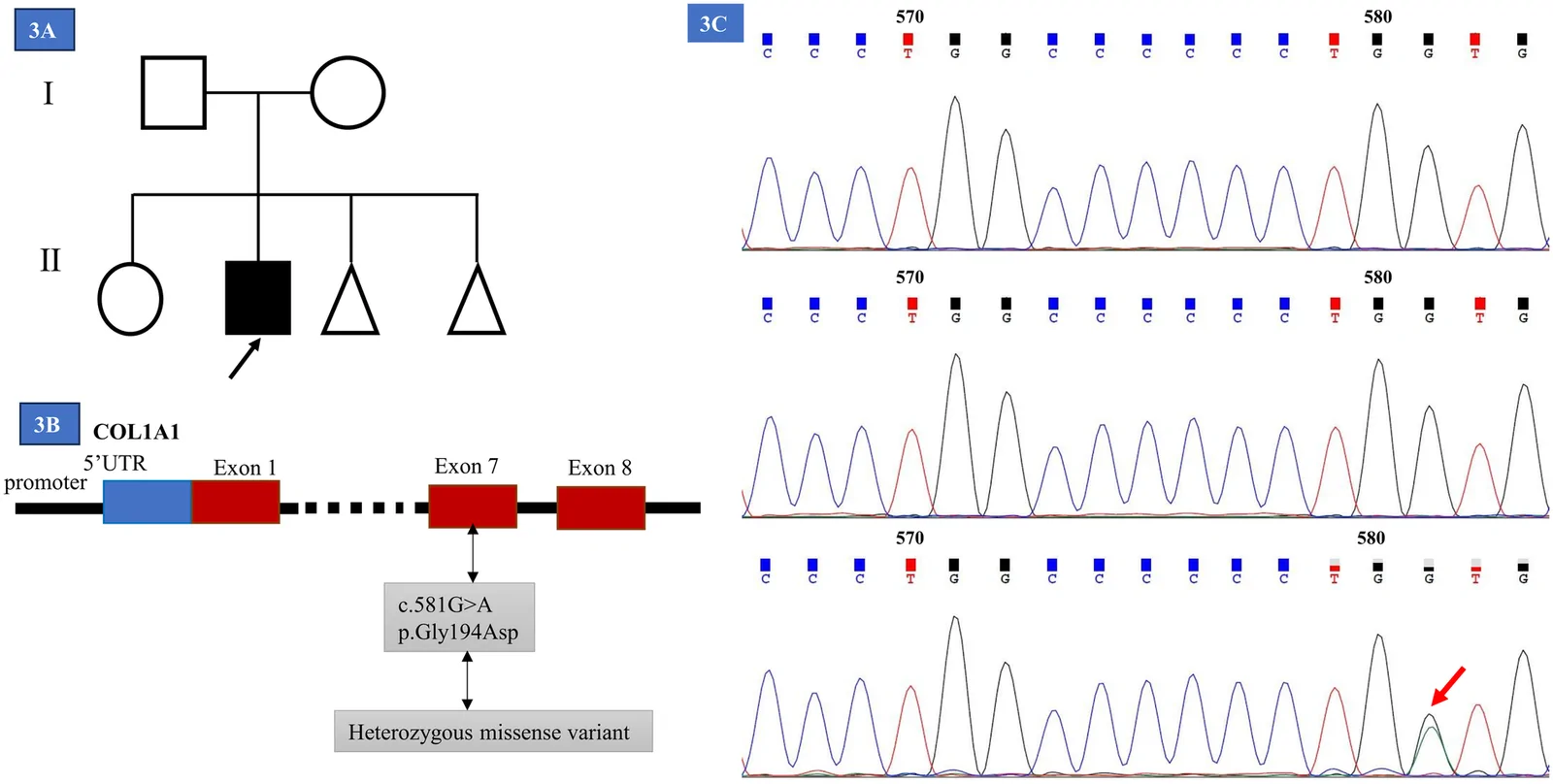
Family pedigree and molecular genetic findings. **(A)** Family pedigree. Both parents were clinically unaffected, and the male infant was the only family member reported to have OI. The two triangles in generation II represent previous pregnancy losses of unknown fetal sex, rather than affected females. Squares denote males, circles denote females, and the filled square denotes the affected infant; the arrow indicates the proband. **(B)** Trio whole-exome sequencing identified a heterozygous missense variant in COL1A1: NM_000088.4:c.581G > A (p.Gly194Asp). **(C)** Sanger sequencing detected the variant in the patient but not either parent, supporting an apparently *de novo* occurrence.

### 2.10 Treatment and clinical course

Following fracture identification, the patient received multidisciplinary management involving neonatology, pediatric orthopedics, radiology, clinical genetics, and nursing specialists.

#### 2.10.1 Orthopedic management

Because of the extreme fragility of the bones, conservative management was selected. Treatment included gentle handling and positioning, avoidance of unnecessary manipulation, fracture stabilization, Pavlik harness fixation for the femoral fracture (Figure 1E), immobilization of the radial fracture. A bone-protective nursing protocol was implemented, including minimal lifting, careful diaper changing, avoidance of excessive limb traction, optimized positioning. No additional fractures were reported during hospitalization. The Pavlik harness was applied on June 1, with four weeks of stabilization initially planned; radial immobilization was documented by June 2.

#### 2.10.2 Respiratory and supportive management

During hospitalization, the infant developed intermittent tachypnea and oxygen desaturation. High-flow nasal cannula (HFNC) support was provided at a flow rate of 3 L/min, with a fraction of inspired oxygen (FiO₂) of approximately 30%. Respiratory status subsequently improved. HFNC began on June 3, and oxygen was discontinued on June 5. A concurrent Staphylococcus haemolyticus bloodstream infection was treated; the relative contributions of rib injury and other illness to respiratory instability cannot be established.

#### 2.10.3 Jaundice management

Phototherapy was administered for neonatal hyperbilirubinemia. The bilirubin concentration gradually decreased, and no additional intervention was required.

### 2.11 Follow-up

The infant was discharged in stable condition on June 11, 2026, with orthopedic review advised one week later. At approximately 3 weeks after discharge, follow-up examination demonstrated improved limb movement and no newly identified fractures. Radiographic evaluation approximately 1 month after discharge showed callus formation, progressive healing of the femoral fracture, and mild residual angulation (Figure 2C). Long-term follow-up was planned, including monitoring of growth and skeletal development, assessment of bone mineral density (BMD) when clinically appropriate, evaluation for recurrent fractures, consideration of intravenous bisphosphonate therapy (pamidronate or zoledronic acid) according to disease severity and clinical evolution (14–18). The overall clinical course, including presentation, diagnostic evaluation, treatment, and follow-up, is summarized in Figure 4.

**Figure 4:**
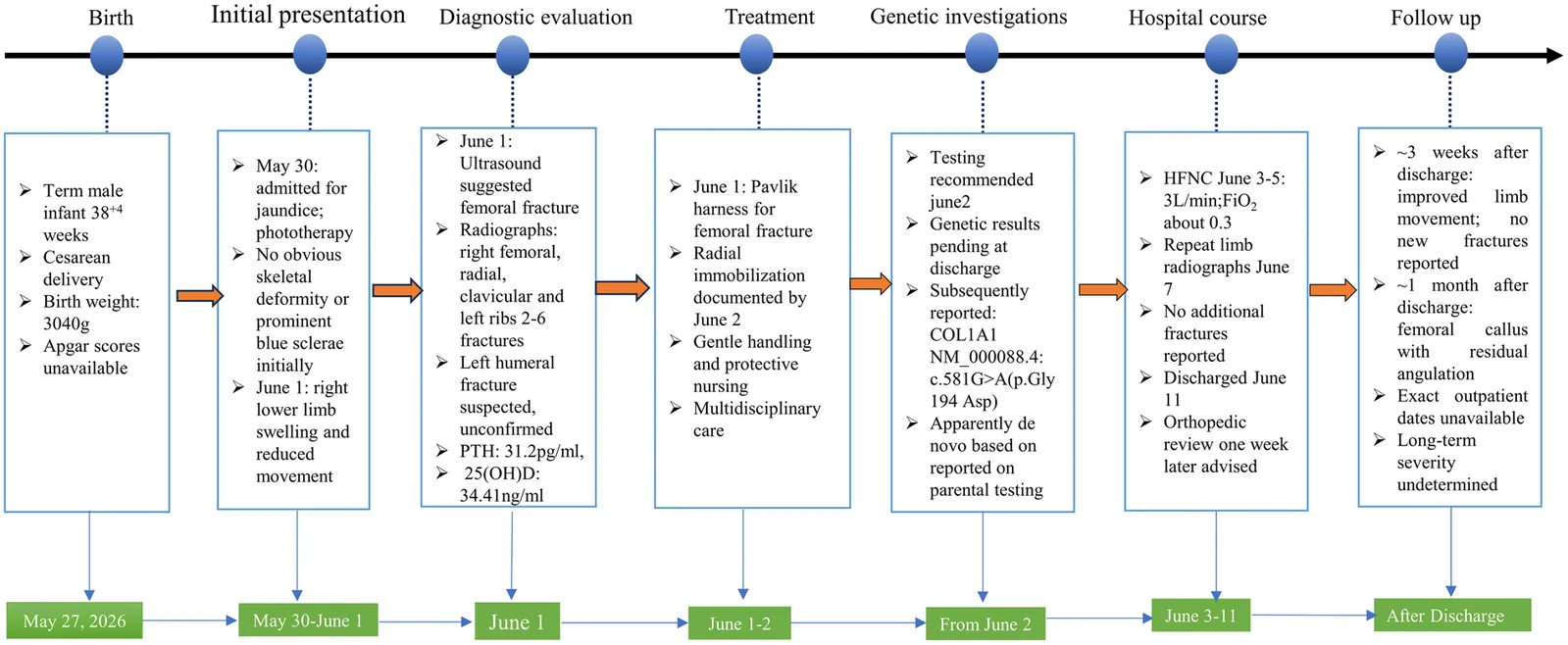
Reported clinical sequence and early follow-up. Birth May 27, 2026, at 38 + 4 weeks, 3040 g; jaundice admission May 30; limb symptoms, ultrasound, and radiographic detection of right femoral, radial, clavicular, and left second to sixth rib fractures June 1; Pavlik harness June 1 and radial immobilization by June 2; genetic testing recommended June 2, with results pending at discharge; HFNC June 3–5; repeat limb imaging June 7; discharge June 11. Clinical and radiographic follow-up were reported approximately three weeks and one month after discharge. Detection dates do not establish fracture occurrence dates.

## 3 Discussion

### 3.1 Diagnostic challenges of neonatal osteogenesis imperfecta without classical manifestations

Osteogenesis imperfecta (OI) is a genetically heterogeneous disorder characterized by impaired skeletal integrity and increased susceptibility to fractures (1–5). Although severe forms, particularly Sillence type III OI, are typically associated with prenatal fractures, skeletal deformities, and progressive bone fragility, early recognition remains challenging in some neonates because classical clinical manifestations may be absent.

In the present case, the infant was initially admitted to the neonatal intensive care unit (NICU) because of progressive jaundice rather than suspected skeletal disease. The diagnosis was considered only after progressive limb swelling and reduced spontaneous movement led to radiological evaluation and identification of multiple fractures. Previous studies have demonstrated that *de novo* variants in COL1A1 and COL1A2 account for a substantial proportion of molecularly confirmed OI cases, meaning that affected infants may have no family history of disease (5, 6, 13). Therefore, neonatologists should maintain a high index of suspicion for inherited skeletal fragility disorders when fractures are detected without an appropriate traumatic explanation, even when typical features of OI are absent.

### 3.2 Comparison with previously reported neonatal OI cases

Most reported neonatal OI cases present with obvious skeletal abnormalities, including fractures detected prenatally or immediately after birth, limb deformities, and generalized skeletal fragility. A recent case report published in Frontiers in Pediatrics described a neonate with severe OI who received early multidisciplinary management involving neonatologists, orthopedic surgeons, and genetic specialists (19). Similar to that report, our case emphasizes the importance of coordinated multidisciplinary care. However, our patient highlights a distinct diagnostic challenge: OI may initially escape clinical recognition during the early neonatal period. This diagnostic sequence suggests that molecular testing should be considered in neonates with unexplained fractures, even when the clinical presentation is atypical.

### 3.3 Differential diagnosis of multiple fractures in neonates

The differential diagnosis and laboratory evaluation are described in the Case Presentation. Elective cesarean delivery does not exclude birth injury, and the absence of baseline imaging prevents precise dating of fractures. A genetic diagnosis supports inherited fragility but does not independently exclude concomitant injury (10, 11).

### 3.4 Molecular diagnosis and significance of the COL1A1 p.Gly194Asp variant

The diagnosis in this patient was established through trio WES, which identified an apparently *de novo* COL1A1 p.Gly194Asp variant. The COL1A1 gene encodes the *α*1 chain of type I collagen, which forms the major structural framework of the bone extracellular matrix (ECM) (1, 7). The identified variant affects a glycine residue located within the collagen triple-helical domain. The collagen triple helix consists of repeated Gly-X-Y sequences, in which glycine is required at every third position because its small molecular size allows tight packing of the three collagen chains (1, 7, 8). Substitution of glycine with a larger amino acid can disrupt triple-helix formation, alter collagen stability, and impair extracellular matrix integrity (8). Considerable genotype–phenotype variability persists among individuals harboring pathogenic collagen variants, and molecular findings should always be interpreted in conjunction with clinical and radiological features rather than being used in isolation to predict disease severity (3, 5, 13). The variant is not claimed to be previously unreported. Its identification supports COL1A1-related OI but does not establish a progressively deforming Sillence type III phenotype during this short follow-up (4).

### 3.5 Radiological evaluation and diagnostic implications

Radiological assessment plays a central role in the diagnosis of neonatal OI (1, 4). In this patient, radiographs provided the first objective evidence of an underlying skeletal fragility disorder by demonstrating fractures involving femur, radius, clavicle, ribs. The distribution, number, and healing characteristics of fractures are important diagnostic clues. Although generalized osteopenia and skeletal deformities may support the diagnosis of severe OI, these findings may not always be prominent in the neonatal period. Therefore, careful interpretation of fracture patterns is essential. A skeletal survey remains valuable in distinguishing inherited bone fragility disorders from traumatic causes of fractures (10, 11). In particular, the combination of radiological evidence and molecular confirmation provides the most reliable diagnostic approach. Radiological evaluation is also important in excluding alternative diagnoses, particularly when non-accidental injury is considered (10, 11). Bedside ultrasound enabled targeted cortical and soft-tissue assessment without transporting the infant, but is operator dependent and limited to the examined region. It cannot replace appropriate radiographs or a clinically indicated skeletal survey, and a negative local scan does not exclude fractures elsewhere.

### 3.6 Multidisciplinary management of neonatal osteogenesis imperfecta

Management of neonatal OI requires a multidisciplinary strategy aimed at preventing additional fractures, promoting fracture healing, minimizing complications, supporting long-term skeletal development (14, 15, 19). Because the underlying collagen abnormality cannot currently be corrected, early management focuses on gentle handling, fracture stabilization, specialized nursing care, orthopedic intervention, genetic counseling. Previous studies have emphasized that infants with severe OI benefit from coordinated care involving neonatologists, orthopedic specialists, rehabilitation teams, and genetic counselors (14, 15, 19). In our patient, conservative fracture management using a Pavlik harness and protective nursing strategies resulted in progressive fracture healing without additional fracture events during hospitalization. This early observation highlights the importance of minimizing mechanical stress and avoiding preventable secondary injuries.

### 3.7 Bisphosphonate therapy and long-term follow-up

Intravenous bisphosphonates, particularly pamidronate and zoledronic acid, have become important therapeutic options for children with moderate-to-severe OI (15–18, 20–22). These treatments increase BMD; evidence for consistent reductions in fracture frequency and improvements in functional outcomes is less conclusive (17). Early intravenous treatment is established specialist practice for infants with severe OI, although the timing of initiation requires individualized assessment (15). Treatment decisions should consider fracture burden, pain, vertebral compression, skeletal deformity, growth, BMD when clinically interpretable, and cardiorespiratory stability (14–18). Before infusion, calcium and vitamin D status and renal function should be assessed, with close monitoring for hypocalcemia, acute-phase reactions, and cardiorespiratory deterioration during initial treatment (15). In the present case, bisphosphonate therapy was deferred pending long-term assessment, with progressive fracture healing and no newly identified fractures during early follow-up. These short-term observations do not establish that treatment is unnecessary; eligibility and timing require reassessment by a pediatric bone specialist. Long-term surveillance remains essential because progressive skeletal deformities, growth impairment, recurrent fractures, and respiratory complications may develop.

### 3.8 Limitations and long-term follow-up

Follow-up extends to approximately one month after discharge, which is insufficient to assess the natural history of OI, progressive skeletal deformity, long-term fracture burden, or treatment effects. Orthopedic review one week after discharge was advised. A proposed longer-term schedule is specialist review approximately every one to three months during infancy, adjusted to clinical severity, with monitoring of growth, skeletal development, pain, limb alignment, recurrent fractures, respiratory status, and motor development. Vertebral assessment and BMD measurement should be considered when clinically appropriate, with prompt reassessment of bisphosphonate eligibility for recurrent fractures or progressive deformity. This schedule describes planned surveillance rather than completed follow-up.

## 4 Conclusion

Neonatal osteogenesis imperfecta (OI) remains a diagnostic challenge when classical manifestations are inconspicuous. In this infant admitted for jaundice, multiple fractures were subsequently detected and a pathogenic COL1A1 p.Gly194Asp variant supported COL1A1-related OI. Fracture occurrence timing and the Sillence subtype remain uncertain. Early recognition enables protective handling, multidisciplinary fracture care, genetic counseling, and individualized long-term follow-up.

## Parent perspective

The infant cannot provide a personal account. A direct parental perspective was not included in this report.

## Data Availability

The datasets presented in this study can be found in online repositories. The names of the repository/repositories and accession number(s) can be found in the article/Supplementary Material.
