# Supplementary material for: Case Report: Neonatal COL1A1-related osteogenesis imperfecta recognized after multiple fractures in an infant admitted for jaundice
Source: Front Pediatr. 2026 Oct 30;14:1962700. doi: 10.3389/fped.2026.1962700 (PMC13759739; doi:10.3389/fped.2026.1962700)
Supplement: Supplementary file 1 [file Table1.docx]

Supplementary Table 1. Reported clinical laboratory and molecular findings

| **Domain** | **Finding** |
| --- | --- |
| **Perinatal characteristics** |  |
| Gestational age | 38^+4^ weeks |
| Birth weight | 3040 g |
| Mode of delivery | Elective cesarean delivery |
| **Initial clinical presentation** |  |
| Reason for NICU admission | Progressive neonatal jaundice |
| Initial skeletal examination | No obvious skeletal deformity or limb abnormality |
| Blue sclerae | Not apparent at initial examination |
| Family history | No family history of recurrent fractures or skeletal disorders |
| **Fracture presentation and imaging** |  |
| Initial clinical abnormality | Swelling and decreased spontaneous movement of the right lower extremity |
| Bedside examination | Apparent angulation of the right upper limb compared with the contralateral side |
| Bedside ultrasonography | Interruption of the right femoral continuity with surrounding subcutaneous soft-tissue and muscular swelling; possible intermuscular hematoma |
| Subsequent radiography | Right femoral shaft fracture |
| Additional fractures | Right radius, right clavicle, and left second to sixth ribs; suspected left humeral involvement |
| **Laboratory evaluation** |  |
| Calcium | 2.2 mmol/L (reference range: 2.1–2.8 mmol/L) |
| Phosphorus | 1.86 mmol/L (reference range: 1.6–2.51 mmol/L) |
| Alkaline phosphatase (ALP) | 281 U/L (reference range: 48–406 U/L) |
| Parathyroid hormone (PTH) | 31.2 pg/mL (reference range: 14–72 pg/mL) |
| 25-Hydroxyvitamin D (25-OH vitamin D) | 34.41 ng/mL (reference range: >30 ng/mL) |
| **Genetic evaluation** |  |
| Genetic test | Trio whole-exome sequencing |
| Genetic variant | *COL1A1* NM_000088.4:c.581G>A (p.Gly194Asp) |
| Variant inheritance | Apparently de novo based on reported parental testing |
| Variant classification | Reported as pathogenic by the testing laboratory; detailed evidence not independently reassessed |
| **Final diagnosis** | COL1A1-related osteogenesis imperfecta; Sillence subtype unassigned |
